# Polymorphisms in *CYP3A5, CYP3A4*, and *ABCB1* genes: implications for calcineurin inhibitors therapy in hematopoietic cell transplantation recipients—a systematic review

**DOI:** 10.3389/fphar.2025.1569353

**Published:** 2025-07-16

**Authors:** Luiz Carlos da Costa-Junior, Daniely Regina Freitas-Alves, Amanda Melo Leite Leão, Hayra de Andrade Vieira Monteiro, Rita de Cássia Barbosa da Silva Tavares, Maria Claudia Rodrigues Moreira, Marília Berlofa Visacri, Teresa de Souza Fernandez, Paulo Caleb Júnior de Lima Santos

**Affiliations:** ^1^ Postgraduate Program in Medical Sciences, Faculdade de Medicina da Universidade de São Paulo (FMUSP), São Paulo, Brazil; ^2^ Bone Marrow Transplant Center, Instituto Nacional de Câncer (INCA), Rio de Janeiro, Brazil; ^3^ Escola Nacional de Saúde Pública Sérgio Arouca, Fundação Oswaldo Cruz (ENSP-FIOCRUZ), Rio de Janeiro, Brazil; ^4^ Escola de Ciências da Saúde, Universidade do Grande Rio (Unigranrio), Duque de Caxias, Brazil; ^5^ Cytogenetic Laboratory, Cell and Gene Therapy Program, Instituto Nacional de Câncer (INCA), Rio de Janeiro, Brazil; ^6^ Clinical Research Division, Instituto Nacional de Câncer (INCA), Rio de Janeiro, Brazil; ^7^ Bone Marrow Transplant Program, Hospital Universitário Clementino Fraga Filho (HUCFF), Universidade Federal do Rio de Janeiro (UFRJ), Rio de Janeiro, Brazil; ^8^ Departament of Pharmacy, Faculdade de Ciências Farmacêuticas, Universidade de São Paulo (FCF-USP), São Paulo, Brazil; ^9^ Department of Pharmacology, Escola Paulista de Medicina, Universidade Federal de São Paulo (EPM-Unifesp), São Paulo, Brazil

**Keywords:** *CYP3A5*, *CYP3A4*, *ABCB1*, calcineurin inhibitors, hematopoietic cell transplantation

## Abstract

This systematic review assessed the impact of *CYP3A5*, *CYP3A4*, and *ABCB1* polymorphisms on the pharmacokinetics and clinical outcomes of calcineurin inhibitors in hematopoietic cell transplantation (HCT) recipients. Following PRISMA 2020 guidelines, the protocol was registered in PROSPERO (CRD42024517094). A comprehensive search in PubMed, BVS, Scopus, Web of Science, Embase, and Cochrane databases (2013–2024) identified observational studies focusing on tacrolimus or cyclosporine and the specified polymorphisms. Studies on non-human subjects, solid organ transplants, pharmacokinetic models, and drug interactions were excluded. Narrative synthesis was employed due to heterogeneity, and study quality was evaluated using the Newcastle–Ottawa Scale (NOS) and STREGA guidelines. Of 301 records, 11 studies met inclusion criteria, predominantly retrospective and involving adult populations, with sample sizes ranging from 20 to 420 HCT recipients from the USA, Japan, and France. Outcomes included drug levels, median concentration/dose (C/D) ratio, therapeutic index, and clinical endpoints such as graft-versus-host disease (GVHD) and acute kidney injury (AKI). CYP3A5*3 (rs776746) significantly influenced tacrolimus levels, C/D ratio, and clinical outcomes, highlighting its potential as a pharmacogenetic biomarker. *CYP3A4* and *ABCB1* polymorphisms demonstrated limited effects on tacrolimus pharmacokinetics and no significant clinical impact. Methodological quality was high, with 55% of studies achieving the maximum NOS score, although gaps in error rates and population modeling were noted. Limitations include variability in outcomes precluding meta-analysis, a small number of studies, particularly on cyclosporine, and insufficient data on *CYP3A4* and *ABCB1*. Further research is necessary to validate findings.

**Systematic Review Registration:**

https://www.crd.york.ac.uk/PROSPERO/view/CRD42024517094, PROSPERO, CRD42024599998.

## 1 Introduction

Graft-versus-host disease (GVHD) after allogeneic hematopoietic cell transplantation (HCT) is associated with morbidity and mortality (Lee and Flowers, 2008; Malard et al., 2023). The most commonly used regimens for prevention of acute GVHD (aGVHD) consist of a combination of a calcineurin inhibitor (CNI), either cyclosporine (CSP) or tacrolimus (TAC), and an antimetabolite methotrexate (Funke et al., 2023; Penack et al., 2020; Storb et al., 1986; Storb et al., 1989). However, the use of these drugs presents major challenges in clinical practice due to their wide interindividual variability in pharmacokinetics, which may lead to frequent dose adjustments, substitution with other immunosuppressants, or even discontinuation. Therefore, successful treatment involves continuous monitoring of plasma levels within a target range (Brunet et al., 2019; Penack et al., 2024) to avoid subtherapeutic concentrations, which may increase the risk of GVHD (Arcuri et al., 2022; Yee et al., 1988), or supratherapeutic doses, which may increase the risk of toxicities (da Silva et al., 2014; Ram et al., 2012).

Regarding the pharmacokinetics of CNIs, both CSP and TAC undergo hepatic biotransformation primarily mediated by CYP3A4 (Cytochrome P450 Family 3 Subfamily A Member 4) and CYP3A5 (Cytochrome P450 Family 3 Subfamily A Member 5) enzymes, with a greater contribution from CYP3A5 to their oxidation and subsequent elimination. In addition, these drugs are also substrates of the P-glycoprotein (Pgp) efflux pump present in various compartments of the body and expressed by the *ABCB1* (ATP-Binding Cassette Subfamily B Member 1) gene (Barbarino et al., 2013; Forsythe and Paterson, 2014; Hesselink, 2003; Iwasaki, 2007). Therefore, these proteins play a central role in the pharmacokinetics of these two immunosuppressants, potentially interfering with their absorption, distribution, biotransformation, and elimination. Genetic polymorphisms in the *CYP3A5* (Khan et al., 2020; Zhang et al., 2017), *CYP3A4* (Abdel-Kahaar et al., 2019; Kong et al., 2023; Wang et al., 2018), and *ABCB1* (Hu et al., 2018; Lee et al., 2015; Oetting et al., 2018; Rotarescu et al., 2024) genes it could affect the expression of these biotransformation and transport proteins, which could contribute to this interindividual variability in CNI plasma levels and consequently, could contribute to different clinical outcomes.

Recent guideline from the Clinical Pharmacogenetics Implementation Consortium (CPIC) provide a comprehensive set of recommendations for pharmacogenetic-guided TAC starting dose prescribing (Birdwell et al., 2015). However, these recommendations are mainly based on experience in solid organ transplant patients, and there is a lack of evidence to support the application of CPIC recommendations to the allogeneic HCT recipient population. Additionally, there is a significant gap in studies on the pharmacogenetics of CSP, and unlike TAC, there are no established guidelines to inform its dosing in clinical practice. Within this context, the objective of this systematic review is to evaluate the impact of polymorphisms in the *CYP3A5*, *CYP3A4*, and *ABCB1* genes on pharmacokinetics and/or clinical outcomes of the CNIs particularly in the population in HCT recipients.

## 2 Methods

This review followed the Preferred Reporting Items for Systematic Reviews and Meta-Analyses statement (PRISMA) 2020 checklist and reporting guideline (Page et al., 2021). The protocol of the systematic review was registered on PROSPERO, which is available at CRD42024517094.

### 2.1 Eligibility criteria

Inclusion criteria were: (1) articles published in English; (2) primary research articles; (3) observational studies on the pharmacogenetics of CNIs (tacrolimus and/or cyclosporine); and (4) studies that evaluated pharmacokinetic-related polymorphisms in the *CYP3A5*, *CYP3A4*, or *ABCB1* genes, based on germline DNA from hematopoietic cell transplant recipients. Exclusion criteria were (1) non-human (animal models or *in vitro*); (2) approach in any type of solid organ transplant; (3) pharmacogenetics focused on a pharmacokinetic model; (4) genetic polymorphisms with a focus on drug interactions; and (5) articles published in non-Roman characters.

### 2.2 Search strategy

A comprehensive literature search was performed to identify relevant studies in the PubMed, BVS, Scopus, Web of Science, Embase and Cochrane databases published from 1 January 2013, to 9 February 2024. The detailed search strategy for all databases can be found in Supplementary Table S1. References found in included studies were screened for potential studies that had not yet been identified. Duplicate studies were excluded from the analysis.

### 2.3 Study selection

The population, exposure, comparator, outcomes, and study design (PECOS) model was used to select potential studies: P (population), HCT recipient; E (exposure) and C (comparator), patients with different genotypes (wild or altered) of polymorphisms in genes related to calcineurin inhibitors (i.e., genes influencing the pharmacokinetics of TAC and/or CSP: *CYP3A5*, *CYP3A4* and *ABCB1*); O (outcomes), alteration in pharmacokinetic parameters and clinical outcomes such as acute GVHD, acute kidney injury (AKI), neurotoxicity, thrombotic microangiopathy (TMA), and transplant-related mortality, among other complications; and S (study design), observational (cohort, case-control, or cross-sectional). Conference abstracts, reviews, books or book chapters, case reports, letters, or trial registry records were excluded.

Two blinded reviewers (LCCJ and DRFA) independently screened the titles and abstracts of citations to identify potentially relevant studies. Full-text articles were retrieved, and the same two reviewers (LCCJ and DRFA) independently reviewed the articles according to the inclusion criteria. The third (HAVM) and fourth (AMLL) reviewer, after discussion, resolved any disagreements or questions. This process was performed using Rayyan (Ouzzani et al., 2016), a web application developed to assist researchers in conducting systematic reviews.

### 2.4 Data extraction

The information extracted for each included study encompassed the author, year of publication, country of origin, number of patients per study, study design, recruitment period, age range, genotyping method, polymorphisms evaluated in each study, including genotype and/or phenotype, outcomes evaluated, main results found, and funding sources/sponsors. Effect size estimates were analyzed based on metrics such as median drug levels, median concentration/dose (C/D) ratio, cumulative incidence of GVHD, AKI and TMA, in addition to the prevalence of the therapeutic index (supra or subtherapeutic), considered the main outcomes evaluated. Statistical significance was set at ≥ 95%, according to the description of each study. The extraction was conducted by two independent reviewers (LCCJ and DRFA) using standardized spreadsheets in Microsoft Excel and disagreements were resolved through discussion with the third (HAVM) and fourth (AMLL) reviewer.

### 2.5 Quality assessment

The assessment of the methodological quality of the studies (risk of bias) was performed using the Newcastle-Ottawa Scale (NOS) (Wells et al., 2024), applied by two independent reviewers (LCCJ and DRFA). Disagreements between the reviewers were resolved through discussion with a third (HAVM) and fourth (AMLL) reviewer. Three primary domains were assessed for each study: selection, comparability, and exposure. The maximum NOS scores for these domains were 4, 2, and 3 stars, respectively, resulting in a maximum possible total score of 9 stars per study. Studies were categorized as high quality (7–9 stars), moderate quality (4–6 stars), or low quality (0–3 stars).

To assess the quality of reporting of genetic association studies, we used the Strengthening the Reporting of Genetic Association (STREGA) guidelines (Little et al., 2009). These guidelines address five main categories: genotyping methods and errors, population stratification, haplotype variation, Hardy-Weinberg equilibrium, and replication. The first category encompasses five items: genotyping platform, error and call rates, batch genotyping, genotyping centers/laboratories, and the number of individuals with successful genotyping. A total of nine items were evaluated. To compare the quality of reporting of the studies, a total score was calculated by assigning one point to each item, with a higher score indicating better quality of reporting of the genetic study (range 0–9). This instrument was applied by two independent reviewers (LCCJ and DRFA), and disagreements were resolved through discussion with a third (HAVM) and fourth (AMLL) reviewer.

### 2.6 Data synthesis

The characteristics of the studies, their main results, and methodological quality were summarized descriptively through a narrative synthesis, supported by structured tables, as the data were too heterogeneous to be pooled.

## 3 Results

### 3.1 Search results

**FIGURE 1:**
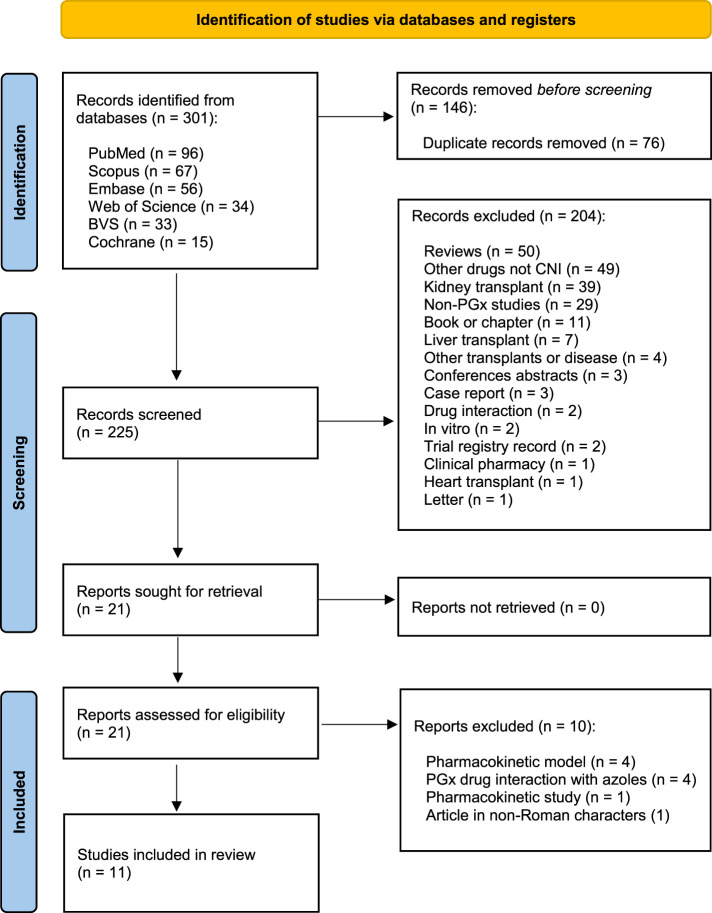
Study selection flowchart through literature search.

The electronic search of the databases resulted in the identification of 301 potentially relevant records. After removing duplicates and initial screening based on titles and abstracts, 21 articles were selected for full-text evaluation. Among the remaining 21 articles, 11 met all the inclusion criteria and did not provide reasons for exclusion. The justification for exclusion as well as the list of excluded articles are available in Supplementary Table S2. The review of the references of the included studies did not reveal any new relevant studies, consolidating the final selection of 11 articles for inclusion in this systematic review (Hamadeh et al., 2019; Ho et al., 2024; Khaled et al., 2016; Laverdière et al., 2015; Pasternak et al., 2022; Seligson et al., 2024; Suetsugu et al., 2019; Thoma et al., 2022; Yamashita et al., 2016; Yoshikawa et al., 2021; Zhu et al., 2020). The study selection process is illustrated in Figure 1.

### 3.2 Study characteristics

The studies included in this systematic review present a diversity of settings, methodological designs, and demographic characteristics of participants, as described in Table 1. The review includes studies conducted in the USA (7), Japan (3), and France (1) with sample sizes ranging from 20 to 420 participants. Study designs include retrospective (n = 7) and prospective (n = 4) approaches. Most studies focused on adult populations, although some included adults and pediatric participants. The CNIs analyzed were predominantly TAC, with only 2 studies investigating CSP. The detailed clinical characteristics of the recipients, donors, and transplants from each of the studies included in this systematic review are available in Supplementary Table S3.

**TABLE 1:** General characteristics of the studies included in systematic review.

Author and year	Country	N	Study design and period	CNI	Age range^a^	Genes	Reference SNP	Pharmacokinetics and Clinical Outcomes	Time Segments for Outcome Evaluation	CNI Therapeutic Range (ng/mL)	Initial CNI Dose (mg/kg/day)
Laverdière, 2015	France	420	Retrospective 1994–2012	CSP	Adult and pediatric	*ABCB1*	(rs1055302, rs2235023, rs4148732, rs6950978)	GVHD acute	100 Days	NI	NI
								GVHD chronic	5 Years		
Khaled et al. (2016)	USA	173	Retrospective 2005–2007	TAC	adult and pediatric	*ABCB1*	(rs1128503, rs2032582, rs1045642, rs3213619)	GVHD acute	100 Days	5–10	0.02
								MAT			
						*CYP3A4*	(rs35599367)	AKI	7, 14 Days		
						*CYP3A5*	(rs776746)	C/D ratio			
								Drug level			
Yamashita et al. (2016)	Japan	24	Prospective 2012–2014	TAC	adult	*CYP3A5*	(rs776746)	GVHD acute	100 Days	10–20	0.03
								AKI			
								Fungal infection			
								Relapse			
								TRM			
								Daily dose			
								Drug level	4-7 Days		
Hamadeh et al. (2019)	USA	63	Retrospective 2016–2017	TAC	adult	*ABCB1*	(rs1128503, rs2032582, rs1045642)	Drug level	Up to 19 Day	5–15	0.03
						*CYP3A4*	(rs2740574, rs35599367)	Sub- or Supratherapeutic level			
						*CYP3A5*	(rs776746, rs14690, rs76293380)	Toxicities			
Suetsugu et al. (2019)	Japan	36	Retrospective 2009–2018	TAC	adult and pediatric	*CYP3A5*	(rs776746)	GVHD acute	28 Days	10–15	0.02–0.03
								AKI			
								C/D ratio	7, 14, 21 Days		
Zhu et al. (2020)	USA	252	Retrospective 2011–2016	TAC	adult	*ABCB1*	(rs1128503, rs2032582, rs1045642)	GVHD acute	100 Days	5–10	0.03
						*CYP3A4*	(rs274057, rs35599367)	AKI	15 Days		
						*CYP3A5*	(rs776746)	Sub- or Supratherapeutic level	3, 6, 9, 12, 15 Days		
								Steady State Concentration			
Yoshikawa et al. (2021)	Japan	20	Prospective 2018–2020	TAC	adult	*CYP3A5*	(rs776746)	C/D ratio	5 Days	NI	NI
Pasternak et al. (2022)	USA	298	Retrospective 2014–2018	TAC	adult and pediatric	*ABCB1*	(rs2032582)	C/D ratio	90 Days	NI	0.03
						*CYP3A4*	(rs2740574, rs138100349)				
						*CYP3A5*	(rs776746, rs10264272)				
Thoma et al. (2022)	USA	43	Prospective NI	TAC and CSP	adult	*CYP3A5*	(rs776746)	Median dose	100 Days	8–12 200–400	0.03 2.5
								therapeutic index			
								Drug level			
Seligson et al. (2024)	USA	103	Prospective 2012–2014	TAC	adult	*ABCB1*	(rs1128503, rs2032582, rs1045642)	GVHD acute	60 Days	8–12	0.03
								AKI			
						*CYP3A4*	(rs2740574, rs35599367)	Neurotoxicity			
						*CYP3A5*	(rs776746)	Drug level			
								Time to therapeutic index			
Ho et al. (2024)	USA	86	Retrospective 2014–2020	TAC	adult	*ABCB1*	(rs1128503, rs2032585, rs1045642)	GVHD acute	100 Days	3–7 10–15	0.01–0.025
								NRM	NI		
						*CYP3A4*	(rs2740574, rs35599367)	OS			
								RFS			
						*CYP3A5*	(rs776746, rs10264272, rs41303343)	Drug level			
								Time to therapeutic index	3 Days		

^a^ Adult ≥18 years.

AKI, acute kidney injury; C/D ratio, Concentration/Dose ratio; CNI, calcineurin inhibitor; CSP, Cyclosporine A; GVHD, Graft-versus-Host Disease; MAT, thrombotic microangiopathy; N, number of participants; NI, not informed; NRM, Non-Relapse Mortality; OS, overall survival; RFS, Relapse-Free Survival; TAC, tacrolimus.

A variety of polymorphisms were examined across the studies, with the *ABCB1* gene presenting the largest number of variants (9), followed by *CYP3A5* (5 variants) and *CYP3A4* (4 variants). Within the *ABCB1* gene, the most widely analyzed polymorphisms were rs1045642, rs1128503 and rs2032582, which were addressed in five studies each. Regarding *CYP3A4*, the most frequently studied polymorphisms were rs2740574 and rs35599367, with 4 and 5 studies, respectively. For the *CYP3A5* gene, the rs776746 polymorphism stands out as the most investigated (10 analyzed studies). Based on the findings of this systematic review, we highlight the role of polymorphisms in the *CYP3A5* gene. The details of all genes and polymorphisms addressed in each of the studies are available in Supplementary Table S4.

Different outcomes were analyzed across the included studies. Clinical outcomes included graft-versus-host disease (GVHD), acute kidney injury (AKI), neurotoxicity, thrombotic microangiopathy (TMA), non-relapse mortality (NRM), overall survival (OS), and relapse-free survival (RFS). Pharmacokinetic outcomes encompassed drug levels, C/D ratio, steady-state concentrations, median dose, and the assessment of subtherapeutic or supratherapeutic ranges (therapeutic index). Additionally, some studies evaluated the time required to reach therapeutic levels.

### 3.3 Methodological quality of systematic reviews

The results of the quality assessment of the studies using the NOS are presented in Table 2. The total score in the studies ranged from six to nine stars. Most of the studies, 55% (6/11), achieved the maximum score (Hamadeh et al., 2019; Ho et al., 2024; Khaled et al., 2016; Laverdière et al., 2015; Pasternak et al., 2022; Zhu et al., 2020), indicating high methodological quality. Meanwhile 18% (2/11) scored eight, demonstrating minor deficiencies in comparability (Seligson et al., 2024) or selection (Suetsugu et al., 2019). Another 18% (2/11) scored 7, both (Yamashita et al., 2016; Yoshikawa et al., 2021) with limitations in representativeness and comparability. Finally, a study with six points (Thoma et al., 2022) presented weaknesses in selection and comparability, representing 9% (1/11) of the total. Overall, the three questions related to the outcome and selection domains were met consistently in all studies, which reinforces the predominance of high-quality studies and increases the reliability of the conclusions presented.

**TABLE 2:** Results of quality assessment using the Newcastle-Ottawa Scale (NOS) for studies.

Author and year	Selection				Comparability	Outcome			Scores
	Representativeness of the exposed cohort	Selection of the non exposed cohort	Ascertainment of exposure	Demonstration that outcome of interest was not present at start of study	Comparability of cohorts on the basis of the design or analysis	Assessment of outcome	Was follow-up long enough for outcomes to occur	Adequacy of follow up of cohorts	
Laverdière et al., 2015	✵	✵	✵	✵	✵✵	✵	✵	✵	9
Khaled et al. (2016)	✵	✵	✵	✵	✵✵	✵	✵	✵	9
Yamashita et al. (2016)		✵	✵	✵	✵	✵	✵	✵	7
Hamadeh et al. (2019)	✵	✵	✵	✵	✵✵	✵	✵	✵	9
Suetsugu et al. (2019)		✵	✵	✵	✵✵	✵	✵	✵	8
Zhu et al. (2020)	✵	✵	✵	✵	✵✵	✵	✵	✵	9
Yoshikawa et al. (2021)		✵	✵	✵	✵	✵	✵	✵	7
Pasternak et al. (2022)	✵	✵	✵	✵	✵✵	✵	✵	✵	9
Thoma et al. (2022)		✵	✵	✵		✵	✵	✵	6
Seligson et al. (2024)	✵	✵	✵	✵	✵	✵	✵	✵	8
Ho et al. (2024)	✵	✵	✵	✵	✵✵	✵	✵	✵	9

Based on the STREGA guidelines, the quality of reporting in genetic studies included in this review is presented in Table 3. The scores of the 11 studies ranged from four to seven, with nine being the maximum possible points. The study conducted by Zhu et al. (2020) attained the highest score (seven), whereas Suetsugu et al. (2019) recorded the lowest score (four).

**TABLE 3:** The quality of reporting using the STrengthening the Reporting of Genetic Association (STREGA) guideline.

Author and year	Description of genotyping methods and errors					Description of modelling population stratification	Description of modelling haplotype variation	Hardy-Weinberg equilibrium was considered	Statement of whether the study is the first report of a genetic association, a replication effort, or both	Total score
	Genotyping methods and platforms	Error rates and call rates	Laboratory/center where the genotyping was done	Genotyping in batches	The number of individuals was successful genotyping					
Laverdière et al., 2015	✓	✓		✓				✓	✓	5
Khaled et al. (2016)	✓	✓		✓	✓		✓		✓	6
Yamashita et al. (2016)	✓			✓	✓			✓	✓	5
Hamadeh et al. (2019)	✓			✓	✓			✓	✓	5
Suetsugu et al. (2019)	✓			✓	✓				✓	4
J. Zhu et al. (2020)	✓	✓		✓	✓	✓		✓	✓	7
Yoshikawa et al. (2021)	✓			✓	✓			✓	✓	5
Pasternak et al. (2022)	✓		✓	✓	✓				✓	5
Thoma et al. (2022)	✓		✓	✓	✓				✓	5
Seligson et al. (2024)	✓			✓	✓			✓	✓	5
Ho et al. (2024)	✓		✓	✓	✓	✓			✓	6

The other 9 studies were divided as follows: 18% (2/11) received a score of six (Ho et al., 2024; Khaled et al., 2016) and 64% (7/11) received a score of five (Hamadeh et al., 2019; Laverdière et al., 2015; Pasternak et al., 2022; Seligson et al., 2024; Thoma et al., 2022; Yamashita et al., 2016; Yoshikawa et al., 2021). The characteristics least reported in the articles were error and call rates, genotyping site, description of population model and haplotype stratification. In contrast, the platform used for genotyping, genotyping in batches, and number of individuals of successful genotyping and replication were reported in all studies. Finally, Hardy-Weinberg equilibrium was considered in six studies (Hamadeh et al., 2019; Laverdière et al., 2015; Seligson et al., 2024; Yamashita et al., 2016; Yoshikawa et al., 2021; Zhu et al., 2020).

### 3.4 Polymorphisms in the *CYP3A5* gene

Except for Laverdière et al., 2015, all articles included in this review evaluated the influence of polymorphisms in *CYP3A5* gene in relation to clinical and/or pharmacokinetic outcomes. The most studied polymorphism was rs776746, cited in ten studies (Hamadeh et al., 2019; Ho et al., 2024; Khaled et al., 2016; Pasternak et al., 2022; Seligson et al., 2024; Suetsugu et al., 2019; Thoma et al., 2022; Yamashita et al., 2016; Yoshikawa et al., 2021; J. Zhu et al., 2020), followed by rs10264272, in two (Hamadeh et al., 2019; Pasternak et al., 2022). A detailed description of all polymorphisms studied in each of the articles is available in Supplementary Table S4.

The results of some studies point to a significant impact of the CYP3A5*3 variant (rs776746) on the pharmacokinetics of TAC. Higher median TAC levels for the homozygous variant group of patients were observed by (Khaled et al., 2016; Seligson et al., 2024; Suetsugu et al., 2019). In addition, higher C/D ratios in the variant group were reported by (Khaled et al., 2016; Pasternak et al., 2022; Yoshikawa et al., 2021). Finally, a higher prevalence of supratherapeutic plasma levels in variant homozygotes was identified by (Khaled et al., 2016; Suetsugu et al., 2019). The details of the main results in each of the studies are presented in Table 4.

**TABLE 4:** Effect of *CYP3A5*, *CYP3A4* and *ABCB1* polymorphisms on pharmacokinetics and clinical outcomes.

Polymorphisms	Genotype or phenotype	Pharmacokinetics and clinical outcomes	Statistic	Reference
Polymorphisms in the *CYP3A5* gene				
CYP3A5*3 (rs776746)	*1/*1 = WT = AA *1/*3 = HT = GA *3/*3 = HV = GG	↑ Median plasma TAC concentration in the first 7 days group *3/*3 (11.0 ng/mL) vs. *1/*1 (9.3 ng/mL) *1/*3 (9.4 ng/mL)	Y	Khaled et al. (2016)
		↑ Median C/D ratio in the first 7 days group *3/*3 (10.6 ng/mL/mg/day) vs. *1/*1 (7.7 ng/mL/mg/day) *1/*3 (8.2 ng/mL/mg/day)	Y	
		↑ Acute GVHD (grade II to IV) Cumulative Incidence 100 days group *1/*1 = 87.5 (17.2–98.9) vs. *1/*3 = 50.0 (33.4–64.4) *3/*3 = 42.6 (33.7–51.2)	Y	
		↓ Incidence of acute GVHD in the *1/*3 group of patients 0.40 (0.19-0.86) and *3/*3 0.32 (0.16-0.61) when compared to the *1/*1 group of patients in multivariate model	Y	
		There was no statistically significant difference between the groups in the cumulative incidence of TMA within 100 days	NS	
		There was no statistically significant difference between the groups in AKI.	NS	
CYP3A5*3 (rs776746)	*1/*1 = WT = AA *3/*3 = HV = GG	↑ Cumulative incidence of acute GVHD (grade III-IV) *1/*1 (36%) vs. *3/*3 (0%)	Y	Yamashita et al. (2016)
		↑ Incidence of AKI in the *3/*3 group (46%) vs. *1/*1 (9%)	Y	
		There was no statistically significant difference in either blood concentrations or daily dose when comparing groups *1/*1 and *3/*3	NS	
		There was no statistically significant difference in *1/*1 and *3/*3 when evaluating TRM, Relapse and Fungal Infection	NS	
CYP3A5*3 (rs776746) CYP3A5*6 (rs14690) CYP3A53*7 (rs76293380)	NM: *1/*1 IM: *1/*3, *1/*6 and *1/*7 PMs: *3/*3, *6/*6 and *7/*7	↑ Prevalence of supratherapeutic plasma concentrations of TAC in the PM group (77.1%) vs. 53.3% in the NM/IM groups	Y	Hamadeh et al. (2019)
		There was no statistically significant difference in median steady-state TAC concentrations between the PM vs. IM/NMs groups	NS	
		TAC-related toxicities did not differ by CYP3A5 phenotypes	NS	
CYP3A5*3 (rs776746)	Group 1 *1/*1 = WT = AA+ *1/*3 = HT = GA Group 2 *3/*3 = HV = GG	↑ Median C/D ratios in the *3/*3 group (12.7 ng/mL/mg/day) when compared to the *1/*1 + *1/*3 group (11.5 ng/mL/mg/day) in the first 1–7-days post-transplant	Y	Suetsugu et al. (2019)
		↑ Median C/D ratios in the *3/*3 group (11.2 ng/mL/mg/day) when compared to the *1/*1 + *1/*3 group (10.0 ng/mL/mg/day) in the first 8-14-days post-transplant	Y	
		↑ Trend in cumulative incidence of acute GVHD (grade II-IV) of *1/*1 + *1/*3 group (36.8%) vs. group *3/*3 (17.6%)	NS	
		There was no statistically significant difference between the 2 groups in AKI.	NS	
CYP3A5*3 (rs776746)	*1/*1 = WT = AA *1/*3 = HT = GA *3/*3 = HV = GG	↑ Steady-state concentration in *3/*3 group (6.2 ng/mL) vs. *1/*1 group patients (2.8 ng/mL)	Y	Zhu et al. (2020)
		↑ Steady-state concentration in *3/*3 group (6.2 ng/mL) vs. *1/*3 group patients (3.0 ng/mL)	Y	
		↑ Median steady-state concentration in the *3/*3 group vs. patients in the *1/*3 and *1/*3 groups up to 15 days post-transplant	Y	
		↓ Time to target trough in group *3/*3 (5.4 days) when compared to groups *1/*3 (7.5 days) and *1/*1 (7.6 days)	Y	
		↑ Odds ratio 7.27 (2.13-33.39) of subtherapeutic dose in the group of patients *1/*1 vs. *3/*3	Y	
		↑ Odds ratio 10.51 (5.27-22.66) of subtherapeutic dose in the group of patients *1/*3 vs. *3/*3	Y	
		There was no statistically significant difference between the groups in AKI.	NS	
		There was no statistically significant difference between the groups in the cumulative of acute GVHD (grade II-IV or III-IV)	NS	
CYP3A5*3 (rs776746)	*1/*3 = HT = GA *3/*3 = HV = GG	↑ C/D ratio in the *3/*3 vs. *1/*3 group on days D+3, D+4 and D+5 post-transplant	Y	Yoshikawa et al. (2021)
CYP3A5*3 (rs776746) CYP3A5*6 (rs10264272)	Expressers: *1/*1, *1/*3, *1/*6 Non-ex-pressers: *3/*3, *3/*6, *6/*6	↑ (20%) C/D ratio on IV TAC in nonexpresser group (589 ng/dL/mg/kg) vs. expresser (492 ng/dL/mg/kg)	Y	Pasternak et al. (2022)
		↓ [(i.v. C/D)/(oral C/D)] ratio in nonexpresser group (2.99) vs. expressers (3.97), adjusted for age and sex	Y	
CYP3A5*3 (rs776746)	*1/*3 = HT = GA *3/*3 = HV = GG	↑ Dose of TAC to achieve therapeutic range (8-12 ng/mL) in group *3/*3 (0.09 mg/kg) vs. *1/*3 (0.02 mg/kg)	Y	Thoma et al. (2022)
		↑ Blood concentrations median of TAC in group *1/*3 (14.3 ng/mL) vs. *3/*3 (11.2 ng/mL)	Y	
		↑ Dose of CSP to achieve therapeutic range in group *1/*3 (5.1 mg/kg) vs. *3/*3 (3.4 mg/kg)	NS	
CYP3A5*3 (rs776746)	Expressers: *1/*1, *1/*3 Non-ex-pressers: *3/*3	↓ 100-day median of plasma TAC concentration in the expresser group when compared to the non-expresser group	Y	Seligson et al. (2024)
		↑ Doses during post-transplant hospitalization D+10 and hospital discharge in the expressed group when compared to the non-expressed group	Y	
		Non-expressors were associated with a reduction in TAC dose while expressors were associated with an increase over the post-transplant period	Y	
		↑ Time to reach therapeutic range of TAC in the expressers group (13.9 days) vs. non-expressers (9.9 days)	Y	
		↑ Longer hospital stays in the expressing group (27.7 days) when compared to the non-expressing group (20.0 days)	Y	
		↓ Frequency of AKI during hospitalization in the expressing group (7.7%) vs. non-expressing group (25%)	Y	
		There was no statistically significant difference for neurotoxicity in relation to the expresser and non-expresser groups	NS	
		There was no statistically significant difference for acute GVHD in relation to the expresser and non-expresser groups	NS	
CYP3A5*3 (rs776746) CYP3A5*6 (rs10264272) CYP3A53*7 (rs41303343)	NM: *1/*1 IM: *1/*3, *1/*6 and *1/*7 PM: *3/*3, *6/*6 and *7/*7	No statistically significant differences were observed between the NM/IM vs. PM groups for prevalence of therapeutic TAC concentrations IV.	NS	Ho et al. (2024)
		↓ NM/IM was less likely to achieve initial therapeutic target concentrations compared with PM (40% NM/IM vs. 76% PM)	Y	
		The median total daily oral TAC dose was slightly higher among NM/IM subjects compared with PM (0.052 mg/kg vs. 0.030 mg/kg)	NS	
		The time to reach therapeutic TAC concentration did not differ between the NM/IM vs. PM groups	NS	
		The cumulative incidence of grade II to IV acute GVHD at day 100 was not significantly different between the NM/IM and PM groups (32% vs. 33%)	NS	
		There was no significant difference in the incidence of moderate to severe chronic GVHD at 36 months between NM/IM and PM (27% vs. 48%)	NS	
		OS, RFS and NRM for NM/IM individuals did not differ from PM.	NS	
Polymorphisms in the *CYP3A4* gene^1^				
*CYP3A4* (rs35599367)	WT = CC HT = CT HV = TT	There was no statistically significant difference between the genotypes and serum TAC level and C/D ratio	NS	Khaled et al. (2016)
		There was no statistically significant difference between the groups in the cumulative incidence of TMA within 100 days	NS	
		There was no statistically significant difference between the groups in AKI.	NS	
		There was no statistically significant difference between the groups in GVHD.	NS	
CYP3A4*1B (rs2740574) CYP3A4*22 (rs35599367)	NM: *1/*1 IM: *1/*22 RM: *1/*1B, *1B/*1B	↑ Prevalence of supratherapeutic TAC concentrations was observed in patients with CYP3A4 IM and NM phenotypes compared to those with the RM phenotype (79.6% vs. 42.9%)	Y	Hamadeh et al. (2019)
		↑ Median TAC concentrations were observed in patients with the CYP3A4 IM/NM phenotype, with a median of 17.1 ng/mL (IQR, 15.0–18.8), compared to 13.9 ng/mL (IQR, 11.2–17.6) in those with the CYP3A4 RM phenotype	Y	
		Tacrolimus-related toxicities did not differ by CYP3A4 phenotypes	NS	
CYP3A4*1B (rs274057) CYP3A4*22 (rs35599367)	WT = AA = *1/*1 HT = AG = *1/*1B HV = GG = *1B/*1B WT = CC = *1/*1 HT = CT = *1/*22 HV = TT = *22/*22	↑ Median steady-state TAC concentrations were observed in patients with at least one CYP3A4*22 allele (8.4 [4.3–14.3] ng/mL) compared to those with the CYP3A41/*1 genotype (5.1 [0.6–27.1] ng/mL)	Y	Zhu et al. (2020)
		↑ longer median time to reach the target trough concentration was observed in the CYP3A4*1/*1B group (7.3 days) compared to the *1/*1 group (5.5 days)	Y	
		↑ longer median time to reach the target trough concentration was observed in CYP3A4*1B/*1B patients (7.9 days) compared to *1/*1 patients (5.5 days)	Y	
		↓ risk of subtherapeutic blood concentrations was observed in *1/*1 patients compared to *1/*1B (OR: 0.21 [0.09-0.48]) and *1B/*1B (OR: 0.18 [0.04-0.60])	Y	
		There was no statistically significant difference between the groups in AKI.	NS	
		There was no statistically significant difference between the groups in the cumulative of acute GVHD (grade II-IV or III-IV)	NS	
		↑ C0/D oral TAC in group *1/*1 (2.93 ng/mL/mg/kg) followed by *1/*1B (2.00 ng/mL/mg/kg) and *1B/*1B (1.35 ng/mL/mg/kg)	Y	
		↑ Blood concentrations of oral TAC in group *1/*1B (5.30 ng/mL) followed by *1B/*1B (3.40 ng/mL)	Y	
CYP3A4*1B (rs2740574) CYP3A4*22 (rs138100349)	NM: *1/*1 PM: *22/*22, 1*B/*22 RM: *1/*1B, *1B/*1B	The i.v. C/D did not differ significantly between CYP3A4 NM and CYP3A4 PM.	NS	Pasternak et al. (2022)
		↑ Median TAC iv C/D ng/dL; mg/kg was observed in the NM group compared to the RM group (592 vs. 475)	Y	
CYP3A4*1B (rs2740574) CYP3A4*22 (rs35599367)	NM: *1/*1 IM: *1/*22 PM: *22/*22 RM: *1/*1B, *1B/*1B	73% of CYP3A4 RMs achieved initial therapeutic goal concentrations compared with 66% of CYP3A4 NM/IM/PMs	NS	Ho et al. (2024)
		A lower proportion of CYP3A4 RM attained initial target goal concentrations following the switch to oral tacrolimus compared with CYP3A4 NM/IM/PM (43% CYP3A4 RM vs. 75% CYP3A4 NM/IM/PM).	NS	
		No significant associations were identified in the median total daily oral tacrolimus dose based on CYP3A4	NS	
		The cumulative incidence of grade II to IV acute GVHD at day 100 was not significantly different between the RM and PM/NM/IM groups (20% vs. 35%)	NS	
		There was no difference in the incidence of moderate to severe chronic GVHD at 36 months among the YP3A4 RM versus CYP3A4 NM/IM/PM (45% vs. 33%)	NS	
		OS, RFS and NRM for PM/IM/RM individuals did not differ from NM.	NS	
Polymorphisms in the *ABCB1* gene				
*ABCB1* rs4148732 rs6950978	WT = AA HT = AG or AT HV = GG or TT	There was no statistically significant association between the polymorphisms and grade II-IV or III-IV acute GVHD.	NS	Laverdière et al., 2015
		↑ Competing risks of death before acute GvHD were associated with SNPs rs4148732 and rs6950978 in the ABCB1 gene	Y	
*ABCB1* rs1045642 rs1128503 rs2032582 rs3213619	WT = CC or AA HT = CT or AG HV = TT or GG	There was no difference between the genotype groups of any of the four polymorphisms evaluated and the TAC levels	NS	Khaled et al. (2016)
		There was no difference between the genotype groups of any of the four polymorphisms evaluated and the levels and the C/D ratio	NS	
		↑ increase in TAC levels was observed in the CT group, with an elevation of 1.08 ng/mL over time compared to the CC and TT groups for rs1128503	Y	
		There was no statistically significant difference between the groups in AKI.	NS	
		There was no statistically significant difference between the groups in the cumulative incidence of TMA within 100 days	NS	
		There was no statistically significant difference between the groups in GVHD.	NS	
*ABCB1* rs1128503 rs2032582 rs1045642	NF = WT IF = HT LF = HV	↑ Prevalence of supratherapeutic concentrations of TAC in the IF or LF groups when compared to NF (86.5% vs. 50%) for ABCB1 C2677T	Y	Hamadeh et al. (2019)
		There was no significant difference in the prevalence of supratherapeutic tacrolimus concentrations between the IF/LF phenotype of ABCB1 C3435T or C1236T (76.2%) and the NF phenotype (61.9%)	NS	
		↑ Median TAC concentration was 17.3 ng/mL (IQR, 15.6–19.0) for patients with the ABCB1 C2677T IF/LF phenotype, compared with 14.9 ng/mL (IQR, 11.6–17.4) in those with the ABCB1 C2677T NF phenotype	Y	
		There were no significant differences in median TAC steady-state concentrations between ABCB1 C3435T IF/LF (17.0 ng/mL; IQR, 14.5–19.0) and ABCB1 C3435T NF (16.0 ng/mL; IQR, 12.1–17.6)	NS	
		There were no significant differences in median TAC steady-state concentrations between ABCB1 C1236T IF/LF (17.0 ng/mL; IQR, 14.5–18.8) and ABCB1 C1236T NF (15.2 ng/mL; IQR, 11.8–17.6)	NS	
		↑ Odds of TAC-related adverse events were observed in the ABCB1 C2677T LF phenotype, with 84.6% compared to the NF phenotype (42.3%)	Y	
		TAC-related adverse events did not differ by ABCB1 C1236T or ABCB1 C3435T phenotypes	NS	
*ABCB1* rs1128503 rs2032582 rs1045642	WT = CC HT = CT HV = TT	No significant differences were detected between all three ABCB1 SNPs and the median TAC trough concentration	NS	Zhu et al. (2020)
		Patients with the C/C genotype for ABCB1 C1236T and C3435T reached target TAC trough concentrations between days +4 and +6, while those with C/T and T/T genotypes reached it between days +1 and +3	NS	
		For ABCB1 C2677T, patients with the C/C genotype had lower target TAC trough concentrations between days +10 and +12, but this association lost significance after adjustment	NS	
		Associations between time to target steady-state TAC trough concentrations by ABCB1 SNPs showed no significant differences for the three ABCB1 variants	NS	
		↑ Odds (OR: 2.08) of subtherapeutic plasma concentrations were observed in patients with the CC genotype compared to the CT group for ABCB1 C1236T	Y	
		↑ Odds (OR: 2.71) of subtherapeutic plasma concentrations were observed in patients with the CC genotype compared to the TT group for ABCB1 C2677T	Y	
		There was no statistically significant difference between the groups in AKI.	NS	
		There was no statistically significant difference between the groups in the cumulative of acute GVHD (grade II-IV or III-IV)	NS	
*ABCB1* rs2032582	WT = GG HT = GT or GA HV = TT or AA	No significant difference in i.v. C/D was found among the ABCB1 genotype groups	NS	Pasternak et al. (2022)
*ABCB1* rs1045642 rs1128503 rs2032582	WT = CC HT = CT HV = TT	There was no statistically significant difference between the genotypes of each polymorphism and the TAC levels	NS	Suetsugu et al. (2019)
*ABCB1* rs1045642 rs1128503 rs2032582	WT = CC or AA HT = CT or AG HV = TT or GG	The prevalence of TAC plasma concentrations, both oral and intravenous, within the therapeutic index did not differ between the genotypes of the polymorphisms	NS	Ho et al. (2024)
		No significant associations were identified between ABCB1 genotype and the total daily dose of iv. TAC at the time therapeutic concentrations were attained	NS	
		OS, RFS and NRM for PM/IM/RM individuals did not differ from NM.	NS	
		There was no statistically significant difference between the groups in the cumulative of acute GVHD.	NS	

1 Seligson et al. (2024) did not detail the results for *CYP3A4*.

↑ (Greater or Higher), ↓ (Lesser or Lower), AKI (Acute Kidney Injury), C/D ratio (Concentration-to-Dose Ratio), CSP (Cyclosporine A), D+ (Day Post-Transplant), GVHD (Graft-Versus-Host Disease), HT (Heterozygous), HV (Homozygous Variant), IM (Intermediate Metabolizer), IF (Intermediate Function), IV (Intravenous), LF (Less Function), NM (Normal Metabolizer), NF (Normal Function), NRM (Non-Relapse Mortality), NS (Not Significant), OS (Overall Survival), PM (Poor Metabolizer), RFS (Relapse-Free Survival), RM (Rapid Metabolizer), TAC (Tacrolimus), TMA (Thrombotic Microangiopathy), TRM (Treatment-Related Mortality), Vs. (Versus), WT (Wild Type), Y (Yes).

Regarding clinical outcomes, two studies suggest the influence of polymorphisms on the incidence of acute GVHD. Khaled et al. (2016) reported a higher incidence of acute GVHD (grades II-IV) in the wild-type group (*1/*1) compared to homozygous variant (*3/*3) patients. Similarly, Yamashita et al. (2016) observed a higher incidence of severe acute GVHD (grades III-IV) in patients in the wild-type group (*1/*1) compared to the homozygous variant (*3/*3) group. On the other hand, four studies (Ho et al., 2024; Seligson et al., 2024; Thoma et al., 2022; J. Zhu et al., 2020) did not identify statistically significant association between the incidence of acute GVHD and polymorphisms.

Regarding the incidence of AKI, homozygous variant genotypes (*3/*3) were associated with a higher frequency, as demonstrated by Yamashita et al. (2016), Seligson et al. (2024). In contrast, four studies (Ho et al., 2024; Khaled et al., 2016; Suetsugu et al., 2019; Zhu et al., 2020) did not identify a statistically significant association between the incidence of AKI and polymorphisms.

### 3.5 Polymorphisms in the *CYP3A4* gene

Four distinct polymorphisms were evaluated in 45% (5/11) of the studies in this review (Hamadeh et al., 2019; Ho et al., 2024; Khaled et al., 2016; Pasternak et al., 2022; Zhu et al., 2020). With citation in five different studies (Hamadeh et al., 2019; Ho et al., 2024; Khaled et al., 2016; Seligson et al., 2024; Zhu et al., 2020) rs35599367 was the most studied, followed by rs2740574 with four (Hamadeh et al., 2019; Ho et al., 2024; Pasternak et al., 2022; Seligson et al., 2024).

Differences in median plasma levels of TAC were observed in two studies. Hamadeh et al. (2019) demonstrated higher concentrations for the CYP3A4 IM/NM phenotype and Zhu et al. (2020) demonstrated higher concentrations for CYP3A4*1/*1B. In contrast, (Khaled et al., 2016; Ho et al., 2024) did not find differences between the groups according to the phenotype. Regarding the optimal therapeutic index of TAC, Hamadeh et al. (2019) demonstrated a higher prevalence of supratherapeutic plasma concentrations of TAC in patients with CYP3A4 IM and NM phenotype. On the other hand, Zhu et al. (2020) demonstrated a lower risk of subtherapeutic contractions for the group of patients with CYP3A4 *1/*1 phenotype.

Regarding the C/D ratio, Zhu et al. (2020) observed that the highest TAC ratio was in the CYP3A4 *1/*1 group, followed by the CYP3A4*1/*1B and CYP3A4*1B/*1B groups. Pasternak et al. (2022), in turn, demonstrated a higher ratio for the CYP3A4 NM phenotype in relation to CYP3A4 RM. In this case, no differences were observed between the CYP3A4 NM and CYP3A4 PM groups. Finally, in the study by Khaled et al. (2016), no significant differences were demonstrated in the C/D ratio.

The influence of polymorphisms in *CYP3A4* on clinical outcomes was evaluated in four studies: AKI in four studies (Hamadeh et al., 2019; Ho et al., 2024; Khaled et al., 2016; Zhu et al., 2020), GVHD in three studies (Ho et al., 2024; Khaled et al., 2016; Suetsugu et al., 2019), TMA in one study (Khaled et al., 2016) and OS, RFS and NRM in one study (Ho et al., 2024). In all the studies reviewed, no statistically significant difference was identified between the groups of different genotypes/phenotypes regarding the occurrence of any of the outcomes mentioned above.

### 3.6 Polymorphisms in the *ABCB1* gene

Nine different polymorphisms for the *ABCB1* gene were evaluated in 63% (7/11) of the studies (Hamadeh et al., 2019; Ho et al., 2024; Khaled et al., 2016; Laverdière et al., 2015; Pasternak et al., 2022; Seligson et al., 2024; Zhu et al., 2020). The three most studied polymorphisms, each with five citations, were rs1045642 and rs1128503, both reported in studies (Hamadeh et al., 2019; Ho et al., 2024; Khaled et al., 2016; Seligson et al., 2024; Suetsugu et al., 2019; Zhu et al., 2020), and rs2032582, reported in studies (Hamadeh et al., 2019; Khaled et al., 2016; Pasternak et al., 2022; Seligson et al., 2024; Zhu et al., 2020). The other six polymorphisms had only one citation.

Among the studies included in this review, only Laverdière et al., 2015 used the CNI CSP for GVHD prophylaxis. They identified that wild-type genotypes for the rs4148732 and rs6950978 polymorphisms of the *ABCB1* gene are associated with a lower competitive risk of death before the development of acute GVHD. However, none of the polymorphisms evaluated increased the incidence of grade II-IV or III-IV acute GVHD. Other studies (Ho et al., 2024; Khaled et al., 2016) that evaluated the risk of GVHD corroborate the lack of influence of polymorphisms in *ABCB1* on the incidence of the disease.

Regarding TAC plasma levels, Khaled et al. (2016) pointed out that the genotype (heterozygous) of rs1128503 is associated with higher levels, while Hamadeh et al. (2019) demonstrated that the heterozygous or homozygous variant genotypes in rs2032582 also result in higher plasma concentrations. Zhu et al. (2020), on the other hand, demonstrated an association of the wild-type (CC) genotype in the rs1128503 and rs2032582 polymorphisms with a higher probability of subtherapeutic plasma concentrations of TAC. Despite this finding, other studies indicate the absence of significant associations between *ABCB1* gene polymorphisms and plasma levels (Ho et al., 2024; Khaled et al., 2016; Seligson et al., 2024), C/D ratio (Khaled et al., 2016; Pasternak et al., 2022).

Finally, regarding the clinical outcomes evaluated in the studies, AKI (Khaled et al., 2016; Seligson et al., 2024; Zhu et al., 2020), TMA (Khaled et al., 2016) or OS, RFS and NRM (Ho et al., 2024) were not influenced by *ABCB1* polymorphisms. Only the study by Hamadeh et al. (2019) highlighted that carriers of homozygous variant genotypes for rs2032582 have a higher risk of toxicity associated with the use of TAC.

## 4 Discussion

This is the first systematic review to investigate the influence of genetic polymorphisms on the pharmacokinetics and/or outcomes of CNIs (TAC and CSA), especially in HCT recipients. Eleven studies were included, mostly retrospective and conducted in the USA, with the majority focusing on TAC, and only two addressing CSA. We highlight the significant impact of the *CYP3A5* rs776746 polymorphism on the pharmacokinetics of TAC, such as TAC levels, C/D ratio or therapeutic index, and on clinical outcomes, such as AKI and GVHD. In contrast, polymorphisms in *CYP3A4* and *ABCB1* showed less consistent results, evidencing a variability in the impact of these genes on pharmacokinetic and clinical parameters.

Previous research on the pharmacogenetics of CNIs has predominantly concentrated on solid organ transplantation populations (Hesselink, 2003; Kreutz et al., 2004; Sun et al., 2017; Zhu et al., 2011). However, the pharmacokinetic dynamics of these drugs in HCT differ substantially from those in solid organ transplant settings, leading to distinct clinical consequences. For example, the effects of GVHD (Przepiorka et al., 1999) and graft-versus-leukemia (GVL) (Arcuri et al., 2022; Kolb, 2008; Sweeney and Vyas, 2019) highlight the complex interplay between plasma drug levels and therapeutic outcomes in HCT recipients.

Regarding the characteristics of the population and transplantation, our study showed a predominance of TAC use compared to CSP. Only two studies (Laverdière et al., 2015; Thoma et al., 2022) reported the use of CSP for GVHD prophylaxis. Currently, there are no specific recommendations in the guidelines (Funke et al., 2023; Penack et al., 2020; Penack et al., 2024) of societies regarding the choice between CNIs. This finding highlights the importance of future studies investigating the use of CSP for GVHD prophylaxis, aiming to evaluate its impact and expand the available evidence.

The studies included in this review that investigated polymorphisms in the *CYP3A5* gene unanimously demonstrated the influence of these polymorphisms on pharmacokinetics and/or clinical outcome mainly related to TAC. The pharmacokinetic alterations were drug level (Khaled et al., 2016; Seligson et al., 2024; Thoma et al., 2022; Yamashita et al., 2016; J. Zhu et al., 2020), C/D ratio (Khaled et al., 2016; Pasternak et al., 2022; Suetsugu et al., 2019; Yoshikawa et al., 2021), and therapeutic index (Hamadeh et al., 2019; Ho et al., 2024; Thoma et al., 2022; Zhu et al., 2020). The main polymorphism associated with the alterations was CYP3A5*3 (rs776746). However, three studies considered another phenotypic classification in addition to CYP3A5*3: (Pasternak et al., 2022) used CYP3A5*3 and CYP3A5*6, and (Hamadeh et al., 2019; Ho et al., 2024) used CYP3A5*3, CYP3A5*6 and CYP3A53*7 to classify HCT recipient.

The variant alleles of CYP3A5 (*3, *6, or *7) may result in a truncated messenger RNA with loss of expression of the functional protein in homozygotes or heterozygotes or encode a nonfunctional protein (Kuehl et al., 2001). Consequently, with a nonfunctional protein, more drugs would accumulate in the body, increasing drug levels, C/D ratio, and increased toxicities. The studies by Yamashita et al. (2016), Seligson et al. (2024) demonstrated an increase in AKI in non-expressing recipient HCT when compared to expressers. On the other hand, patients classified as expressers, with an efficient performance in drug biotransformation, may contribute to lower plasma levels. The studies by Khaled et al. (2016), Yamashita et al. (2016) demonstrated a higher incidence of acute GVHD in the CYP3A5*1 expresser group when compared to the CYP3A5*3 non-expresser group.

Regarding *CYP3A4* gene polymorphisms, among the five studies analyzed, three demonstrated the influence of these polymorphisms on the pharmacokinetics of TAC. The associations were in relation to drug level, therapeutic index (Hamadeh et al., 2019; Zhu et al., 2020) and C/D ratio (Pasternak et al., 2022; Zhu et al., 2020). Of these three studies, (Pasternak et al., 2022; Hamadeh et al., 2019) used the phenotypic classification of the groups CYP3A4*1B and CYP3A4*22, while Zhu et al. (2020) evaluated the genotypes of each polymorphism separately. None of the studies that evaluated polymorphisms in *CYP3A4* found associations with any clinical outcome. A possible explanation for this finding is the fact that CYP3A4 plays a supporting role in the biotransformation of TAC (Barbarino et al., 2013). Therefore, a reduction in the expression of this enzyme would not be able to cause the same impact as non-expressers of CYP3A5.

Regarding polymorphisms of the *ABCB1* gene, of the seven studies that investigated these variants, four identified associations with the pharmacokinetics and/or clinical outcomes of TAC. The alterations *c.1236C>T* (rs1128503) and *c.2677G>A* (rs1128503) were the two most cited with associations with drug level (Hamadeh et al., 2019; Khaled et al., 2016), therapeutic index (Hamadeh et al., 2019; Zhu et al., 2020) and/or toxicity (Hamadeh et al., 2019). High levels of TAC were associated with alterations in heterozygosity or homozygosity of both polymorphisms. The polymorphism *c.2677G>A* (rs1128503) is a non-synonymous SNP, which causes the substitution of the amino acid alanine for serine or threonine at position 893 of P-glycoprotein, reducing the expression of the transport protein (Gréen et al., 2006; Kim, 2001). The *c.1236C>T* (rs1128503) is a silent polymorphism, and despite not having an amino acid change, changes in mRNA stability, in the amount of translated protein and in the function of the transporter are observed (Kimchi-Sarfaty et al., 2007). The reduction in expression and/or protein with low function could justify the accumulation of the drug in the body and consequently in plasma levels, which would increase the risk of adverse events, which was demonstrated by Hamadeh et al. (2019).

This systematic review has some limitations that should be considered when interpreting the results. The wide variability of outcomes reported in the studies made it impossible to perform a meta-analysis, reducing the ability to integrate the findings quantitatively. In addition, the relatively low number of articles available on the subject, especially those addressing the use of CSP, limits the generalization of the results. The lack of evaluation of polymorphisms in the *ABCB1* and *CYP3A4* genes in some of the studies limits the interpretations related to these genetic markers. Another limitation refers to the small number of patients in some of the included studies, which reduces the statistical power of the analyses and may compromise the robustness of the findings. The restriction to articles published in Roman characters may have resulted in the exclusion of relevant studies written in non-Roman characters, potentially limiting the comprehensiveness of the review. Finally, the long interval between the search strategy and the conduction of the analyses may have excluded relevant studies published later, impacting the timeliness and comprehensiveness of the data reviewed.

## 5 Conclusion

Based on the results of this systematic review, we highlight the role of polymorphisms in the *CYP3A5* gene, especially CYP3A5*3 (rs776746), as a potential predictive biomarker for pharmacokinetic alterations of TAC in HCT recipients. The studies indicated the influence of this polymorphism on plasma levels, C/D ratio, therapeutic index and, in some cases, clinical outcomes. In contrast, polymorphisms investigated in the *CYP3A4* and *ABCB1* genes demonstrated a modest impact on the pharmacokinetics of TAC, being evaluated in a limited number of studies, like what occurs with CSP. These limitations suggest the need for new well-designed clinical studies, with adequate sample size, methodology and results described in detail, focused on the investigation of pharmacogenetics.

## Data Availability

The original contributions presented in the study are included in the article/Supplementary Material, further inquiries can be directed to the corresponding authors.
