# Supplementary material for: Polymorphisms in CYP3A5, CYP3A4, and ABCB1 genes: implications for calcineurin inhibitors therapy in hematopoietic cell transplantation recipients—a systematic review
Source: Front Pharmacol. 2025 Jul 16;16:1569353. doi: 10.3389/fphar.2025.1569353 (PMC12309307; doi:10.3389/fphar.2025.1569353)
Supplement: Supplementary file 1 [file Table1.docx]

| Supplementary Table 1. Search strategy used in each database | |
| --- | --- |
| **Databases** | Database search date: February 9, 2024 |
|  | Period filter: From 2013 to 2024 |
| PUBMED  *All Fields* | "Polymorphism, Genetic" OR "Gene Polymorphism" OR "Gene Polymorphisms" OR "Genetic Polymorphism" OR "Genetic Polymorphisms" OR "Polymorphism (Genetics)" OR "Polymorphism, Gene" OR "Polymorphisms (Genetics)" OR "Polymorphisms, Gene" OR "Polymorphisms, Genetic" OR pharmacogenomics OR pharmacogenomic OR "pharmacogenetic drug" OR pharmacogenetic OR "pharmacogenetic testing" OR "clinical pharmacogenetics" OR "clinical pharmacogenetic"  AND  Tacrolimus OR "Anhydrous Tacrolimus" OR "Anhydrous, Tacrolimus" OR "FK 506" OR FK-506 OR FK506 OR "FR 900506" OR "FR-900506" OR FR900506 OR Prograf OR Prograft OR "Tacrolimus Anhydrous" OR "Tacrolimus, Anhydrous" OR Cyclosporine OR Ciclosporin OR CsA Neoral OR CsA-Neoral OR CsANeoral OR CyA NOF OR CyA-NOF OR Cyclosporin OR "Cyclosporin A" OR "Cyclosporine A" OR Neoral OR "OL 27 400" OR "OL 27-400" OR "OL 27400" OR Sandimmun OR "Sandimmun Neoral" OR Sandimmune OR "Calcineurin Inhibitors" OR "Calcineurin Antagonists" OR "Calcineurin Blockers" OR "Calcineurin Inhibitor" OR "Inhibitor, Calcineurin" OR "Protein Phosphatase 2B Inhibitors" OR "Protein Phosphatase 3 Inhibitors" OR "Protein Phosphatase-2B Inhibitors"  AND  "Bone Marrow Transplantation" OR "Bone Marrow Cell Transplantation" OR "Bone Marrow Grafting" OR "Grafting, Bone Marrow" OR "Transplantation, Bone Marrow" OR "Transplantation, Bone Marrow Cell" OR "Transplantation, Homologous" OR "Allogeneic Grafting" OR "Allogeneic Transplantation" OR Allografting OR "Grafting, Allogeneic" OR Homografting OR "Homologous Transplantation" OR "Transplantation, Allogeneic" OR "Hematopoietic Stem Cell Transplantation" OR "Stem Cell Transplantation, Hematopoietic" OR "Transplantation, Hematopoietic Stem Cell" OR "Cell Transplantation" OR "hematopoietic stem cell transplantation" OR "Hematopoietic Cell Transplantation" OR "stem cell transplantation" OR "stem cell transplantation, hematopoietic" OR "allogeneic transplantation" OR "allogeneic transplantations" OR "Bone Marrow Cell Transplant" OR "Hematopoietic Cell Transplantation" |
| BVS  *Title, abstract and subject* |  |
| SCOPUS  *All Fields* |  |
| WEB OF SCIENCE  *All Fields* |  |
| EMBASE  *All Fields* |  |
| COCHRANE  *All Fields* |  |

| **Supplementary Table 2.** Reasons for Exclusion of Studies with Corresponding Authors, Year, Titles, and References. | | | |
| --- | --- | --- | --- |
| **Reason for exclusion** | **Authors, year** | **Title** | **Reference** |
|  |  |  |  |
| Pharmacokinetic model  (n = 4) | Feng, 2023 | Initial dosage optimization of cyclosporine in Chinese pediatrics patients undergoing allogeneic hematopoietic stem cell transplantation based on population pharmacokinetics: A retrospective study. | BMJ Paediatrics Open. 7(1):e002003 |
|  |  |  |  |
|  | Kim, 2015 | Population pharmacokinetics of cyclosporine in hematopoietic stem cell transplant patients: consideration of genetic polymorphisms. | Ann Pharmacother. 49(6):622-630 |
|  |  |  |  |
|  | Li, 2019 | Population pharmacokinetics of cyclosporine in Chinese children receiving hematopoietic stem cell transplantation. | Acta Pharmacol Sin 40(12):1603-1610 |
|  |  |  |  |
|  | Xue, 2014 | Population pharmacokinetics and individualized dosage prediction of cyclosporine in allogeneic hematopoietic stem cell transplant patients. | Am J Med Sci. 348(6):448-454 |
|  |  |  |  |
|  |  |  |  |
| Pharmacogenetics (PGx) drug interaction with azoles  (n = 4) | Iwamoto, 2015 | Effect of Genetic Polymorphism of *CYP3A5* and *CYP2C19* and Concomitant Use of Voriconazole on Blood Tacrolimus Concentration in Patients Receiving Hematopoietic Stem Cell Transplantation. | Ther Drug Monit. 37(5):581-588. |
|  |  |  |  |
|  | Nara, 2013 | Effect of itraconazole on the concentrations of tacrolimus and cyclosporine in the blood of patients receiving allogeneic hematopoietic stem cell transplants. | Eur J Clin Pharmacol. 69(6):1321-1329 |
|  |  |  |  |
|  | Zeng, 2020 | Effect of cyclosporine and polymorphisms in CYP2C19 and ABCC2 on the concentration of voriconazole in patients undergoing allogeneic hematopoietic stem cell transplantation. | Xenobiotica. 50(5):614-619 |
|  |  |  |  |
|  | Zgheib, 2020 | The role of candidate genetic polymorphisms in the interaction between voriconazole and cyclosporine in patients undergoing allogeneic hematopoietic cell transplantation: An explorative study. | Curr Res Transl Med. 68(2):51-58. |
|  |  |  |  |
|  |  |  |  |
| Pharmacokinetic study  (n = 1) | Li, 2021 | Influential Factors and Efficacy Analysis of Tacrolimus Concentration After Allogeneic Hematopoietic Stem Cell Transplantation in Children with β-Thalassemia Major. | Pharmgenomics Pers Med. 24:14:1221-1237 |
|  |  |  |  |
|  |  |  |  |
|  |  |  |  |
| Article in non-Roman characters (n = 1) | Chen, 2021 | The effect of CYP3A5 gene polymorphism on tacrolimus concentration and adverse events in patients undergoing allogeneic hematopoietic stem cell transplantation. | Chinese J Hematology. 42(10):828–833 |
|  |  |  |  |

| **Supplementary Table 3.** Clinical Characteristics of Recipients, Donors, and Transplants of the Studies Included in the Systematic Review. | | | | | | | | | | | | |
| --- | --- | --- | --- | --- | --- | --- | --- | --- | --- | --- | --- | --- |
| **Authors,**  **Year** | **Country** | **N** | **CNI** | **Race** | **Sex**  (M/F) | **Age**  (median, CI or SD) | | **Donor type** | **HSC source**  (BM/PB/UCB) | **Disease** | **GVHD prophylaxis** | **Conditioning** |
|  |  |  |  |  |  |  |  |  |  |  |  |  |
| Laverdière, (2014) | France | 420 | CSP | NR | 242/178 | <20: 146  20-50: 253  >50: 26 | Adult and pediatric | RD: 241  URD: 179 | 269/151/0 | NMH: 98  MH: 322 | CSP+MTX | MA: 394  NMA: 26 |
|  |  |  |  |  |  |  |  |  |  |  |  |  |
|  |  |  |  |  |  |  |  |  |  |  |  |  |
| Khaled,  2016 | USA | 173 | TAC | W: 91  H: 52  A: 23  O: 7 | 93/80 | 46 (10-70) | Adult and pediatric | RD: 82  URD: 91 | 20/153/0 | AML: 63  ALL: 39  NHL: 23  MDS: 18  CML: 9  MPD: 9  HL: 8  MM: 4 | TAC+Siro  MTX+ATG | Flu+Mel: 103  FTBI+Cy: 9  FTBI+VP-16: 46  Bu+Cy: 15 |
|  |  |  |  |  |  |  |  |  |  |  |  |  |
|  |  |  |  |  |  |  |  |  |  |  |  |  |
| Yamashita, 2016 | Japan | 24 | TAC | NR | 17/7 | 39 (21-63)  55 (36-65) | Adult | RD: 18  URD: 2  O: 4 | 18/2/4 | AML  or MDS: 19  ALL: 5 | MTX  MMF  MTX+MMF | RIC: 10  MA:14 |
|  |  |  |  |  |  |  |  |  |  |  |  |  |
|  |  |  |  |  |  |  |  |  |  |  |  |  |
| Hamadeh, 2019 | USA | 63 | TAC | C: 46  AA: 14  O: 3 | 41/22 | 61 (25-78) | Adult | RD: 17  Haplo: 46 | NR | ALL: 8  AML: 27  CML: 5  LH/NHL:10  MDS: 10  O: 3 | TAC+Cy | Flu+CY+TBI: 61  Bu+Cy: 2 |
|  |  |  |  |  |  |  |  |  |  |  |  |  |
|  |  |  |  |  |  |  |  |  |  |  |  |  |
| Suetsugu, 2019 | Japan | 36 | TAC | NR | 21/15 | 55 (34-69)  55 (17-67) | Adult and pediatric | RD: 8  URD: 28 | 19/10/7 | AML/MDS:20  ALL:3  CML:2  LH/LNH:10  AA:1 | MTX  MMF  mPSL  MMF+CY | MA: 12  RIC: 24 |
|  |  |  |  |  |  |  |  |  |  |  |  |  |
|  |  |  |  |  |  |  |  |  |  |  |  |  |
| Zhu,  2020 | USA | 252 | TAC | W: 211  B: 30  O: 11 | 145/107 | 52 (19-76) | Adult | RD: 165  URD: 87 | 14/237/1 | AA: 5  AL: 139  CL: 16  LH/LNH: 33  MDS/MPS:56  MM=3 | NR | MA: 131  RIC: 121 |
|  |  |  |  |  |  |  |  |  |  |  |  |  |
|  |  |  |  |  |  |  |  |  |  |  |  |  |
| Yoshikawa, 2021 | Japan | 20 | TAC | NR | 13/7 | 55 (38-63)  61 (38-98) | Adult | NR | 13/3/4 | AML: 4  ALL: 3  CML: 2  MDS: 4  NHL: 1  ATL: 4  DLBCL: 1  MF: 1 | TAC+MTX: 15  TAC+MMF: 4  PTCy+TAC+MMF:1 | Flu+Mel+TBI: 11  Cy+TBI: 5  Bu+Cy: 4 |
|  |  |  |  |  |  |  |  |  |  |  |  |  |
|  |  |  |  |  |  |  |  |  |  |  |  |  |
| Pasternak, 2022 | USA | 298 | TAC | C: 275  AA: 14  O: 9 | 171/127 | 47.3 ± 20.1 | Adult and pediatric | NR | NR | NR | TAC: 298 | NR |
|  |  |  |  |  |  |  |  |  |  |  |  |  |
|  |  |  |  |  |  |  |  |  |  |  |  |  |
| Thoma, 2022 | USA | 43 | TAC  and  CSP | W: 41  I: 2 | 32/11 | 61 (25-70) | Adult | RD: 22  URD: 17  Haplo: 4 | 4/37/2 | ALL: 6  AML: 21  CLL: 1  MF: 1  MDS: 9  SCN: 1  CMML: 4 | MTX+TAC or CSP | Flu+Mel: 19  Bu+Cy: 2  Flu+TBI: 3  Flu+Bu: 5  Flu+Cy+TBI: 4  Flu+Mel+TBI: 5  Cy+TBI: 4  Imob+Flu+TBI: 1 |
|  |  |  |  |  |  |  |  |  |  |  |  |  |
|  |  |  |  |  |  |  |  |  |  |  |  |  |
| Seligson, 2023 | USA | 103 | TAC | EA: 88  NEA: 15 | 64/39 | 55 (18-81) | Adult | RD: 35  URD: 55  O: 13 | 2/88/13 | AML: 41  ALL: 8  NHL: 8  MF: 7  Other: 39 | TAC | MA: 13  RIC: 90 |
|  |  |  |  |  |  |  |  |  |  |  |  |  |
|  |  |  |  |  |  |  |  |  |  |  |  |  |
| Ho, 2024 | USA | 86 | TAC | W: 70  B: 7  O: 9 | 51/35 | 57 (20-74) | Adult | RD: 86 | 4/82/0 | AA: 3  ALL: 12  AML: 36  CML: 2  MDS/MPS: 20  MM: 2  LNH/CLL: 11 | TAC+MTX: 17  TAC+MMF: 5  TAC+Siro: 48 | MA: 53  RIC: 33 |
|  |  |  |  |  |  |  |  |  |  |  |  |  |
| **AA**: African American, **A**: Asiatic, **ALL**: Acute Lymphoblastic Leukemia, **AML**: Acute Myeloid Leukemia, **ATL**: Adult T-cell Leukemia, **ATG**: Anti-thymocyte globulin, **B**: Black, **BM**: Bone Marrow, **Bu**: Busulfan, **C**: Caucasian, **CI**: Confidence Interval, **CLL**: Chronic Lymphocytic Leukemia, **CML**: Chronic Myeloid Leukemia, **CMML**: Chronic Myelomonocytic Leukemia, **CNI**: Calcineurin inhibitor, **CSP**: Cyclosporine, **Cy**: Cyclophosphamide, **DLBCL**: Diffuse Large B-Cell Lymphoma, **EA**: European ancestry, **Flu**: Fludarabine, **GVHD**: Graft-versus-host disease, **H**: Hispanic, **Haplo**: Haploidentical donor, **HCT**: Hematopoietic Cell Transplantation, **HL**: Hodgkin Lymphoma, **HSC**: Hematopoietic Stem Cell, **I**: Indian, **Imob**: Mobilized, **MA**: Myeloablative conditioning, **MDS**: Myelodysplastic Syndrome, **Mel**: Melphalan, **MF**: Myelofibrosis, **MM**: Multiple Myeloma, **MMF**: Mycophenolate Mofetil, **MPD**: Myeloproliferative Disorders, **MTX**: Methotrexate, **N**: Number, **NEA**: Non-European ancestry, **NHL**: Non-Hodgkin Lymphoma, **NMA**: Non-myeloablative conditioning, **NR**: Not reported, **O**: Other, **PB**: Peripheral Blood, **PTCy**: Post-Transplant Cyclophosphamide, **RD**: Related Donor, **RIC**: Reduced Intensity Conditioning, **SD**: Standard Deviation, **Siro**: Sirolimus, **SCN**: Severe Congenital Neutropenia, **TAC**: Tacrolimus, **TBI**: Total Body Irradiation, **UCB**: Umbilical Cord Blood **URD**: Unrelated Donor, **VP-16**: Etoposide, **W**: White. | | | | | | | | | | | | |

| **Supplementary Table 4.** Distribution of polymorphisms in the *CYP3A5*, *CYP3A4* and *ABCB1* genes by author and year. | | | | | | | | | | | | |
| --- | --- | --- | --- | --- | --- | --- | --- | --- | --- | --- | --- | --- |
| **Genes and**  **SNP** | **Author and year of publication** | | | | | | | | | | | **All** |
|  | Hamadeh, 2019 | Ho,  2024 | Khaled, 2016 | Laverdière, 2014 | Pasternak, 2022 | Seligson, 2023 | Suetsugu, 2019 | Thoma,  2022 | Yamashita, 2016 | Yoshikawa, 2021 | Zhu,  2020 |  |
|  |  |  |  |  |  |  |  |  |  |  |  |  |
| ***CYP3A5*** | **✔** | **✔** | **✔** |  | **✔** | **✔** | **✔** | **✔** | **✔** | **✔** | **✔** | **10** |
| rs10264272 |  | **✔** |  |  | **✔** |  |  |  |  |  |  | 2 |
| rs14690 | **✔** |  |  |  |  |  |  |  |  |  |  | 1 |
| rs41303343 |  | **✔** |  |  |  |  |  |  |  |  |  | 1 |
| rs76293380 | **✔** |  |  |  |  |  |  |  |  |  |  | 1 |
| rs776746 | **✔** | **✔** | **✔** |  | **✔** | **✔** | **✔** | **✔** | **✔** | **✔** | **✔** | 10 |
|  |  |  |  |  |  |  |  |  |  |  |  |  |
|  |  |  |  |  |  |  |  |  |  |  |  |  |
| ***CYP3A4*** | **✔** | **✔** | **✔** |  | **✔** | **✔** |  |  |  |  | **✔** | **6** |
| rs138100349 |  |  |  |  | **✔** |  |  |  |  |  |  | 1 |
| rs274057 |  |  |  |  |  |  |  |  |  |  | **✔** | 1 |
| rs2740574 | **✔** | **✔** |  |  | **✔** | **✔** |  |  |  |  |  | 4 |
| rs35599367 | **✔** | **✔** | **✔** |  |  | **✔** |  |  |  |  | **✔** | 5 |
|  |  |  |  |  |  |  |  |  |  |  |  |  |
|  |  |  |  |  |  |  |  |  |  |  |  |  |
| ***ABCB1*** | **✔** | **✔** | **✔** | **✔** | **✔** | **✔** |  |  |  |  | **✔** | **7** |
| rs1045642 | **✔** | **✔** | **✔** |  |  | **✔** |  |  |  |  | **✔** | 5 |
| rs1055302 |  |  |  | **✔** |  |  |  |  |  |  |  | 1 |
| rs1128503 | **✔** | **✔** | **✔** |  |  | **✔** |  |  |  |  | **✔** | 5 |
| rs2032582 | **✔** |  | **✔** |  | **✔** | **✔** |  |  |  |  | **✔** | 5 |
| rs2032585 |  | **✔** |  |  |  |  |  |  |  |  |  | 1 |
| rs2235023 |  |  |  | **✔** |  |  |  |  |  |  |  | 1 |
| rs3213619 |  |  | **✔** |  |  |  |  |  |  |  |  | 1 |
| rs4148732 |  |  |  | **✔** |  |  |  |  |  |  |  | 1 |
| rs6950978 |  |  |  | **✔** |  |  |  |  |  |  |  | 1 |
